# Trunk Impairment Scale and Stroke Impact Scale for Clinical Assessment of Patients in the Subacute Stage After Stroke Following Sensory Intervention

**DOI:** 10.33549/physiolres.935710

**Published:** 2025-12-01

**Authors:** Martin GÁBOR, Diana BZDÚŠKOVÁ, Jana KIMIJANOVÁ, Zuzana HIRJAKOVÁ, Helena ŠINGLIAROVÁ, Peter VALKOVIČ

**Affiliations:** 1 2nd Department of Neurology, Faculty of Medicine, Comenius University Bratislava, Bratislava, Slovakia; 2 Department of Physical Therapy and Rehabilitation, University Hospital Bratislava, Bratislava, Slovakia; 3 Department of Behavioural Neuroscience, Institute of Normal and Pathological Physiology, Centre of Experimental Medicine, Slovak Academy of Sciences, Bratislava, Slovakia

**Keywords:** Stroke, Trunk impairment scale, Stroke impact scale, Postural balance, Sitting

## Abstract

Stroke survivors frequently present with impaired trunk control, which is a key determinant of mobility, balance, and independence in activities of daily living (ADL). Reliable clinical tools are therefore needed to evaluate postural stability, particularly in patients unable to stand. This randomized controlled study assessed the applicability of the Trunk Impairment Scale (TIS) and Stroke Impact Scale (SIS) in post-stroke patients after completion of a complementary sensory intervention targeting sitting postural stability. Forty inpatients in the subacute stage after stroke were randomized into an Experimental group receiving daily postural training with visual biofeedback in addition to standard physiotherapy, and a Control group receiving standard physiotherapy only. Assessments included TIS and SIS at baseline and post-intervention. Both groups demonstrated significant improvements in trunk control, mobility, strength and ADL over time, as reflected by higher TIS and SIS scores. However, the Experimental group achieved greater gains, with the most pronounced effects observed in TIS, as well as SIS Mobility, and SIS ADL domains. Mobility improvements were strongly associated with enhanced ADL performance, underscoring the relevance of trunk control rehabilitation. These findings confirm the clinical sensitivity of TIS and SIS in capturing meaningful postural changes associated with functional recovery after stroke. This study demonstrates that targeted trunk-focused interventions with complementary sensory input can significantly enhance both motor and functional outcomes in stroke survivors. Combining TIS and SIS provides a comprehensive evaluation of clinical performance and patient-reported outcomes, offering valuable insight for rehabilitation strategies aimed at improving independence and quality of life.

## Introduction

Stroke is among the leading causes of mortality and long-term disability worldwide [1]. Despite significant advances in acute management, a substantial proportion of stroke survivors remain dependent in daily activities and experience persistent motor and cognitive impairments [2]. Impaired postural control is one of the most disabling sequelae, directly limiting functional recovery and increasing the risk of falls [3,4]. Falls are frequent in the post-stroke population, occurring in 50–70 % of survivors, and they lead to secondary complications such as fractures, fear of falling, and reduced social participation [5]. Thus, the socioeconomic burden of stroke arises not only from mortality but also from chronic disability, reduced quality of life, and the long-term need for rehabilitation [1,6].

Postural control requires the integration of visual, vestibular, and somatosensory information, as well as coordinated activation of trunk musculature [7]. Damage to cortical and subcortical structures after stroke disrupts these interactions, leading to impaired trunk performance, asymmetrical weight distribution, and altered spatial body representation [8,9]. Patients with right hemispheric lesions in particular often exhibit more pronounced postural asymmetry, neglect, and verticality misperception [10]. These impairments manifest not only in standing but also in sitting, which constitutes a fundamental prerequisite for gait recovery and functional independence [11]. Importantly, sitting balance has been shown to predict mobility, walking independence, and discharge outcomes, even in the subacute phase when many patients are unable to stand safely [12,13].

Trunk performance is a critical determinant of functional prognosis after stroke. Restoration of trunk control is strongly associated with improvements in balance, gait, and independence in activities of daily living (ADL) [14,15]. Several systematic reviews have confirmed that targeted trunk training can enhance sitting and standing balance as well as mobility outcomes [16]. Therefore, accurate assessment of trunk performance is essential not only for documenting baseline impairments but also for guiding rehabilitation interventions and monitoring progress.

To capture these aspects, specific clinical assessment tools have been developed. The Trunk Impairment Scale (TIS) is a standardized measure that evaluates static and dynamic sitting balance as well as trunk coordination. It has demonstrated excellent inter-rater and test–retest reliability, strong validity, and sensitivity to change during rehabilitation [17]. By quantifying trunk performance across three subscales, TIS provides valuable information for clinicians about the quality of trunk movement and its contribution to overall postural control. The Stroke Impact Scale (SIS), on the other hand, is a comprehensive, stroke-specific, self-reported outcome measure assessing eight domains, including strength, ADL, mobility, hand function, memory, communication, emotion, and participation [18–20]. SIS was developed with direct input from patients and caregivers, ensuring that it captures the multidimensional consequences of stroke on health-related quality of life. By incorporating both physical and psychosocial aspects, SIS complements performance-based instruments such as TIS and provides insight into how impairments affect daily life from the patient’s perspective.

Although both instruments are widely used in stroke rehabilitation research and practice, evidence regarding their combined application in patients with severe motor deficits and limited standing capacity remains scarce. In particular, patients who are unable to stand safely are frequently excluded from studies of balance and postural stability, despite representing a large and clinically important subgroup [21]. Previous research on trunk training and interventions using visual biofeedback has highlighted the crucial role of proximal stability in facilitating distal mobility and gait recovery [22,23]. Furthermore, disturbances in verticality perception, egocentric and allocentric reference frames, and spatial body representation after stroke have been shown to contribute to postural asymmetry and instability [24,25]. These findings underscore the need for reliable clinical measures that can be applied in sitting and that capture both functional performance and perceived impact.

Integrating scales such as TIS with patient-reported outcomes such as SIS may therefore provide a more comprehensive evaluation of sitting postural control and functional state after stroke. This combined approach offers the opportunity to relate clinical impairments of trunk function to broader domains of participation and quality of life. Moreover, it may improve the sensitivity of outcome assessment in intervention studies, particularly those focusing on sitting postural stability, which remains an underexplored area of stroke rehabilitation research.

Therefore, the aim of this study was to examine the clinical applicability of the TIS and SIS in assessing postural stability in subacute post-stroke patients unable to stand. In addition, we investigated whether both clinical scales would show significant changes over time and whether patients receiving a complementary sensory intervention alongside standard rehabilitation would show different gains as a result of the sensory training.

## Methods

### Participants

Forty post-stroke patients from the Department of Physical Therapy and Rehabilitation at the University Hospital Bratislava, Slovakia, participated in the study (descriptive sample statistics are provided in Table 1). The cohort consisted of individuals in subacute stage with residual neurological deficits who were admitted for inpatient rehabilitation.

*Inclusion criteria* for participation were age between 18 and 85 years; confirmed first-ever ischemic or hemorrhagic stroke verified by computed tomography and/or magnetic resonance imaging; subacute stage of stroke (from 1 week to 3–6 months post-onset); presence of residual hemiparesis; and voluntary recruitment. Participants were additionally required to be able to sit without trunk support at least 30 seconds. All patients underwent a doctor–patient medical consultation to validate the inclusion criteria, after which they were evaluated by a physician specialized in rehabilitation medicine who was responsible for supervising participant enrollment and carrying out evaluations using clinical scales.

*Exclusion criteria* were age >85 years, inability to maintain unsupported sitting for at least 30 seconds; neglect syndrome; severe sensory deficits after stroke; polyneuropathy; peripheral neuropathy; communication problems that prevented cooperation or absence of informed consent; neurodegenerative disease; unstable medical condition; severe musculoskeletal injury; contraindication for stimulation intolerance; cognitive impairment defined as MMSE<23 or MoCA<23 that could interfere with comprehension of instruction.

The final cohort comprised 40 patients with a mean age of 65.0 ± 11.1 years (range 35–83), including both sexes and spanning middle-aged to older adults. The majority experienced an ischemic stroke (n = 31; 77.5%), while a smaller proportion had a hemorrhagic stroke (n = 9; 22.5%), reflecting the typical epidemiological distribution of stroke subtypes. The mean time from stroke onset to study enrollment was 16.5 days, with a median of 13 days (interquartile range 11–18), corresponding to the subacute stage. Lesion locations included the basal ganglia, cortical fronto-parietal-temporal-occipital regions, internal capsule, pons, medulla, cerebellum, and thalamus. Lesions were observed in both hemispheres, with hemiparesis evenly distributed between the left (n = 20) and right (n = 20) sides. This balanced distribution enhances the generalizability of the results to a wide range of stroke patients with diverse lesion locations.

The study was approved by the Ethics Committee of the University Hospital Bratislava, Slovakia. All patients provided written informed consent in accordance with the Helsinki Declaration of 1964 (2013 revision) before study enrollment. The trial was registered with the ISRCTN registry (ISRCTN10459491).

### General procedure

This study was part of a broader investigation focused on effects of post-stroke rehabilitation (project APVV-20-0420), specifically targeting the restoration of postural control and body symmetry through sensory intervention. Therefore, only a brief summary of the relevant procedures is provided here. During hospitalization, all recruited patients underwent a baseline assessment (*PRE*) consisting of: i) clinical scales (TIS, SIS) enabling evaluation of both objective and subjective aspects of functional status; and ii) stabilometric assessment of postural stability, including basic sitting balance with eyes open and closed, and maximal voluntary body lean in the frontal plane while sitting. Patients were randomly assigned to either the *Experimental* (n = 20) or *Contro*l (n = 20) group using the sealed envelope method. We ensured a balanced number of patients in both groups, and they did not significantly differ in age, height and weight. All participants from both groups received the standard post-stroke rehabilitation. In addition, the *Experimental* group participated in a complementary sensory intervention consisting of voluntary trunk tilt training in a sitting position, using visual biofeedback based on center of pressure displacement, administered either alone or in combination with unilateral vibration of musculus quadratus lumborum. The intervention lasted for eight days, with one 30-minute training session per day. Upon completion of the intervention (in case of *Experimental* group), or after 8 days of standard rehabilitation (in case of *Control* group), all patients underwent a final assessment (*POST*) using the same clinical scales and stabilometric measures employed during the baseline evaluation. Please note that the primary results on the effects of this intervention are not presented in this paper.

### Clinical scales

Two standardized scales - the Trunk Impairment Scale and the Stroke Impact Scale were used. The SIS, which assesses the impact of stroke on various aspects of health and daily functioning, was administered by a physiotherapist through patient interviews. The TIS was conducted as a direct bedside examination by the physiotherapist. The combined use of a performance-based measure (TIS) and a patient-reported outcome measure (SIS) allowed us to capture complementary perspectives on functional status and the perceived impact of stroke.

The TIS was applied according to the original methodology described by Verheyden *et al.* [13,14,17]. This scale provides a structured evaluation of three domains: static sitting balance, dynamic sitting balance, and trunk coordination (full version of scale is available in Verheyden *et al.* [13]). For static sitting balance, the patient is asked to sit without foot support and with arms resting freely. The examiner observes the ability to maintain this unsupported position, the quality of postural reactions to unexpected perturbations, and the capacity to restore trunk alignment after displacement. Dynamic sitting balance is evaluated by asking the patient to perform controlled lateral and forward weight shifts of the trunk and trunk rotations. The examiner assesses the quality, amplitude, and symmetry of these movements. Finally, trunk coordination is examined through dissociated movements of the upper and lower trunk, such as shoulder rotation against a fixed pelvis or active leg movements with a stabilized trunk. These tasks are designed to reflect the patient’s ability to selectively activate trunk musculature, which is often impaired after stroke. Each item of the TIS is scored on a scale from 0–2 or 0–−3 depending on the task. The maximum total score is 23 points, with higher values representing better trunk performance and greater postural stability. The assessment typically requires 10–15 minutes to complete and can be performed at the bedside without specialized equipment, making it feasible in both inpatient and outpatient settings. The TIS has demonstrated excellent inter-rater and test–retest reliability, strong construct and concurrent validity, and high responsiveness to rehabilitation-induced changes [13].

The SIS version 3.0 was used according to the validation studies by Duncan *et al.* [15,16] and summarized by Mulder and Nijland [18]. The SIS is a comprehensive, stroke-specific, self-report questionnaire that evaluates the impact of stroke across eight domains: strength, memory and thinking, emotion, communication, activities of daily living/instrumental activities of daily living (ADL/IADL), mobility, hand function, and participation (for more details, please see full version in Supplementary Fig. 1). These domains reflect both physical and psychosocial consequences of stroke, thereby providing a multidimensional profile of recovery. Each item is rated on a 5-point Likert scale (1 = extremely difficult, 2 = quite difficult, 3 = somewhat difficult, 4 = little difficult, 5 = no difficulty). Scores for each domain are transformed to a 0–100 scale using the following formula: *Domain score = (mean item score − 1) ÷ 4 × 100.*

The SIS also includes a visual analogue scale (0–100) in which patients rate their overall perceived recovery since the onset of stroke. Completion of the questionnaire requires approximately 15–20 minutes. The instrument does not require formal training, and its administration is straightforward. The SIS has demonstrated excellent psychometric properties, including internal consistency (Cronbach’s α = 0.80–0.95), satisfactory test–retest reliability, and strong concurrent validity compared with other outcome measures such as the Barthel Index, Functional Independence Measure, modified Rankin Scale, and SF-36 [15,26].

### Statistical analysis

The collected data were analyzed in JASP software (JASP Team, version 0.19.1, 2024). Prior to statistical analysis, normality of each scale score distributions were examined using the Shapiro–Wilk test, which showed normal distribution. To examine the effects of the rehabilitation time and the intervention, a repeated measures ANOVA was conducted with *time* (*PRE* vs. *POST* intervention) as the within-subject factor and *group* (*Experimental vs. Control*) as the between-subject factor. This design allowed us to test for the main effect of time (changes across time), the main effect of *group*, and the *time × group* interaction, which reflects whether the change over time differed between the *Experimental* and *Control* groups. Also, the partial eta squared (η^2^p) was computed to estimate effects sizes. The p-values for pair-wise contrasts were corrected with Holm adjustment to account for family-wise error rate (adjusted p-values are reported). Finally, the change scores (Δ) from clinical scales administered *PRE vs. POST* intervention in each group were assessed using correlation analysis. Normality of the change scores distributions was assessed using the Shapiro–Wilk test, which revealed deviations from normality. Therefore, Spearman’s rank correlation coefficients were used to assess associations between changes in outcome measures within the *Experimental* and *Control* groups. Correlation matrices were visualized using heatmaps, with coefficients color-coded according to their magnitude and direction. Statistical significance is indicated by asterisks as follows *p<0.05, **p<0.01, ***p<0.001, except for diagonal values (self-correlations).

## Results

**Fig. 1:**
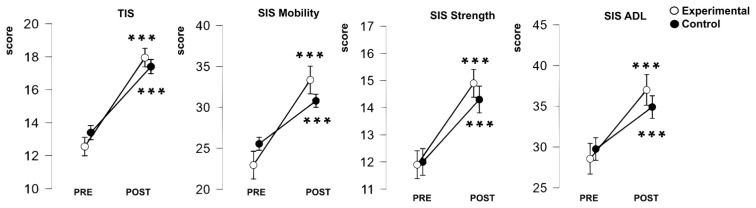
Graphical representation of changes in TIS, SIS Mobility, SIS Strength, and SIS ADL from PRE to POST intervention measurements in the Experimental (white open circles) and the Control (black filled circles) group. Data are presented as mean ± standard error of the mean (SEM). Asterisks indicate significant changes between PRE and POST intervention measurements within each group (***p<0.001).

All outcome measures showed significant improvements over time (*p*<0.001), reflecting a robust *PRE vs. POST* intervention effect (Table 2). However, the extent of improvement differed between groups for several outcomes. Significant *time × group* interactions were observed for TIS, SIS Mobility, and SIS ADL, with the *Experimental* group achieving greater gains than the *Control* group (Fig. 1). In contrast, SIS Strength showed improvement over time in both groups without a significant interaction. No main effect of *group* was found for any scale.

To sum up, the *Experimental* group showed more pronounced improvements than the *Control* group in TIS, SIS Mobility, and SIS ADL, underscoring the specific benefits of the complementary sensory intervention.

**Fig. 2:**
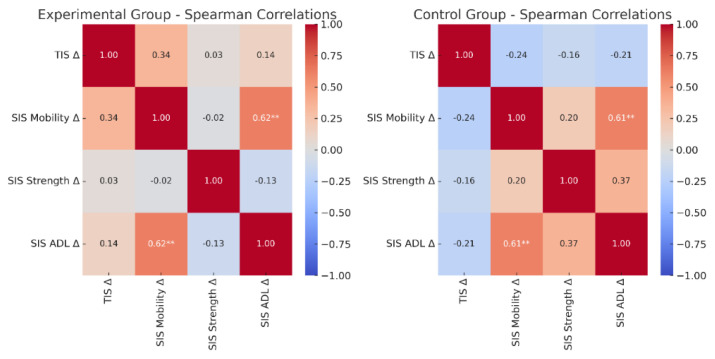
Heatmaps of Spearman’s rank correlation coefficients for changes in clinical scales scores (Δ): TIS, SIS Mobility, SIS Strength, and SIS ADL in the *Experimental* (left) and the *Control* (right) group. Colors represent the strength and direction of the associations (red = positive, blue = negative). Values inside the cells are correlation coefficients; asterisks indicate statistical significance: **p<0.01.

To further examine associations between clinical outcomes, Spearman’s rank correlations were calculated for change scores (Δ). Figure 2 illustrates the correlation heatmaps. In the *Experimental* group, improvements in SIS Mobility were significantly correlated with improvements in SIS ADL (ρ = 0.618, p = 0.004). In the *Control* group, significant positive correlations were observed between SIS Mobility and SIS ADL (ρ = 0.614, *p* = 0.004). In addition, there was a positive trend between change in SIS Strength and change in SIS ADL (ρ = 0.367, p = 0.111), although this did not reach significance. All remaining correlations were weak and nonsignificant.

## Discussion

The aim of this study was to examine the clinical applicability of the TIS and SIS for assessing postural stability in post-stroke patients in the subacute stage unable to stand. A specific aim was to determine whether these clinical scales would improve significantly over time and whether patients receiving a complementary sensory intervention in addition to standard rehabilitation would show greater progress. Our results fulfilled both aims: the clinical scales – TIS and SIS subscales improved significantly during inpatient rehabilitation, consistent with the expected course of recovery. While improvements were evident in both groups, the *Experimental* group demonstrated greater gains in TIS, SIS Mobility, and SIS ADL compared with the *Control* group (Fig. 1), indicating also the specific benefits of the complementary sensory intervention.

The repeated measures ANOVA confirmed a significant effect of *time* across all scales (Table 2), reflecting natural recovery processes in the subacute post-stroke period. No significant main effect of *group* was observed. However, we found the strongest effects in trunk stability (TIS) and functional mobility (SIS Mobility), measures in which a significant *time × group* interaction was detected (Table 2). A smaller but significant interaction effect was also present for SIS ADL. Furthermore, correlation analyses supported these results by highlighting the functional link between trunk control, mobility, and daily activities. The strong positive associations between improvements in SIS Mobility and SIS ADL in both groups (Fig. 2) underscored the functional relevance of mobility recovery for everyday life. Taken together, our results highlight the feasibility of TIS and SIS for evaluating rehabilitation-related progress in subacute post-stroke patients, reinforce previous evidence on the central role of trunk and mobility training, and support integrating targeted sensory interventions into clinical practice as an added value for enhancing functional outcomes.

Our findings are consistent with previous literature [19,20,22,27] and build on long-standing work validating and applying assessment scales in stroke rehabilitation. The Trunk Impairment Scale has repeatedly been described as a valid and reliable tool for assessing postural control [13–14,17]. A recent systematic review [27] confirmed its high sensitivity to change and applicability in both clinical and research contexts. Likewise, the Stroke Impact Scale has been established as a robust instrument capturing patients’ perception of mobility, strength, activities of daily living, and psychosocial functioning [2,15,16,18,28]. Guidetti *et al.* [28] further demonstrated that changes in SIS are clinically meaningful within the first year after stroke, with the strongest effects observed in participation and mobility. This study extends previous findings by showing that both SIS and TIS are feasible and sensitive tools for capturing changes even earlier, during subacute stage (from the first week to approximately three to six months post-stroke). This stage represents a critical window for intensive rehabilitation, when the brain is most responsive to recovery and capable of functional reorganization. During this time, patients typically engage in structured therapy across inpatient, skilled nursing, or home-based settings to improve physical, cognitive, and communication abilities [29,30].

It has been consistently shown that improvements in trunk function and mobility are closely associated with better functional recovery after stroke. For example, Verheyden *et al.* [31] reported that TIS is a strong predictor of independence in activities of daily living, particularly as measured by the Barthel Index at 6 months post-stroke. Kim *et al.* [32] provided further evidence that initial trunk performance predicts subsequent functional outcomes. Likewise, Hsieh *et al.* [33] demonstrated that early trunk control strongly correlates with later ADL function. Studies using the SIS have highlighted mobility as one of the strongest determinants of quality of life and participation [34,35]. Our finding that gains in SIS Mobility correlated with improvements in SIS ADL is consistent with previous reports and confirms that enhanced mobility contributes directly to greater independence in everyday tasks, underscoring its functional relevance during rehabilitation. The absence of a significant *time × group* interaction for SIS Strength suggests that these domains may require longer interventions or additional therapeutic strategies to achieve differential effects. Overall, the present results reinforce the evidence that targeting trunk control and mobility is critical for optimizing functional outcomes in post-stroke rehabilitation.

Our results are consistent also with intervention studies that highlighted the impact of trunk-focused rehabilitation. Haruyama *et al.* [36] demonstrated that core stability training significantly improved gait and balance, Van Criekinge *et al.* [22] confirmed the benefits of robot-assisted trunk training, and Inoue *et al.* [20] showed that biofeedback-based interventions promoted stability improvements. Furthermore, Inoue *et al.* [37] demonstrated that delayed visual feedback during dynamic sitting exercise can enhance postural control in the early post-stroke stage, which aligns with the rationale for our intervention. More recently, additional trials have emphasized novel approaches such as core stabilization combined with conventional therapy [38], trunk motor imagery training [39], and “waist as the axis” therapy [40], all of which reported significant improvements in trunk control and functional balance. To summarize, these studies underscored the potential of targeted interventions to enhance trunk control and functional outcomes. Our findings of significantly improved clinical scale outcomes further demonstrate that visual biofeedback can be effective even in patients unable to stand independently. The strongest *time × group* interactions, observed for TIS, SIS Mobility, and SIS ADL, indicate that the complementary sensory intervention promoted greater improvements beyond standard rehabilitation. These results provide preliminary evidence that visual biofeedback training combined with trunk muscle vibration may enhance recovery of postural stability and functional mobility more effectively than conventional therapy alone by establishing functional links between clinical outcomes and objective postural control.

To conclude, this study demonstrated that the Trunk Impairment Scale and the Stroke Impact Scale are feasible and sensitive clinical tools for assessing changes in subacute post-stroke patients, including those unable to stand. Our findings showed significant improvements over time on both scales and indicated that conventional rehabilitation supplemented with complementary sensory training produced greater gains in postural stability, mobility, and daily activities than standard therapy alone.

### Clinical implications

Sitting balance emerged as a key predictor of functional recovery after stroke. Both the TIS and SIS proved to be feasible instruments for routine use and for monitoring progress over time in subacute post-stroke patients. The TIS captures motor aspects of trunk control, whereas the SIS provides valuable patient-reported perspectives on mobility and quality of life. Visual biofeedback represents a relatively simple method that can be easily integrated into inpatient rehabilitation practice, and recent scoping evidence further supports its relevance in balance training for stroke survivors [41]. Clinically, our findings emphasize the importance of incorporating trunk stability training into standard rehabilitation protocols. The consistent improvements in TIS and SIS Mobility, together with their association with ADL performance, suggest that early targeted interventions focusing on trunk stability may accelerate recovery and promote independence.

### Limitations

Several limitations must be acknowledged. The sample size (n=40) was relatively small, limiting generalizability. The intervention period was restricted to inpatient hospitalization, without long-term follow-up to assess effect sustainability. Finally, this was a single-center study, which may affect external validity. Future studies with larger cohorts and longer follow-up are warranted to confirm these results and explore the sustainability of functional improvements over time.

## Conclusion

In conclusion, this study provides evidence that complementary training targeting trunk stability with visual biofeedback yields greater improvements in postural stability and functional mobility than standard therapy alone. Both the TIS and SIS proved to be suitable scales for monitoring the training-related changes. These findings expand current knowledge on the clinical application of outcome measures and rehabilitation strategies in patients with subacute stroke.

## Supplementary Information



## Figures and Tables

**Table 1:** Basic characteristics of post-stroke patients in both groups.

	Experimental	Control
*Age (years)*	64.1 ± 8.6	65.9 ± 13.3
*Gender (male/female)*	17/3	16/4
*Height (cm)*	175.2 ± 8.3	173.4 ± 11.2
*Weight (kg)*	85.4 ± 16.4	82.4 ± 12.2

Mean ± SD

**Table 2:** Summary of the repeated measures ANOVA outcomes for clinical scales: TIS, SIS (domains: Mobility, Strength, ADL). The models included the effect of time (within-subject factor: PRE and POST), group (between-subject factor: Experimental vs. Control group) and their interaction. Significant effects are bolded.

Scale	Effect	df	F	p	η^2^_p_
*TIS*	Time	1.38	386.829	**< 0.001**	0.911
	Group	1.38	0.030	0.863	0.000
	Time × Group	1.38	8.581	**0.006**	0.184
*SIS Mobility*	Time	1.38	153.950	**< 0.001**	0.802
	Group	1.38	0.0002	0.989	0.000
	Time × Group	1.38	16.671	**< 0.001**	0.305
*SIS Strength*	Time	1.38	121.023	**< 0.001**	0.761
	Group	1.38	0.119	0.732	0.003
	Time × Group	1.38	2.111	0.154	0.053
*SIS ADL*	Time	1.38	74.336	**< 0.001**	0.662
	Group	1.38	0.044	0.836	0.001
	Time × Group	1.38	4.377	**0.043**	0.103
