# Supplementary material for: Trunk Impairment Scale and Stroke Impact Scale for Clinical Assessment of Patients in the Subacute Stage After Stroke Following Sensory Intervention
Source: Physiol Res. 2025 Dec 1;74(Suppl 2):S293–302. doi: 10.33549/physiolres.935710 (PMC12849785; doi:10.33549/physiolres.935710)
Supplement: Supplementary file 1 [file PR74_S293_Suppl_Fig1.pdf]

Stroke Impact Scale

VERSION 3.0

The purpose of this questionnaire is to evaluate how stroke has impacted your health and life. We want to know from **<u>YOUR POINT OF VIEW</u>** how stroke has affected you. We will ask you questions about impairments and disabilities caused by your stroke, as well as how stroke has affected your quality of life. Finally, we will ask you to rate how much you think you have recovered from your stroke.

Stroke Impact Scale

These questions are about the physical problems which may have occurred as a result of your stroke.

**1. In the past week, how would you rate the strength of your....**	**A lot of strength**	**Quite a bit of strength**	**Some strength**	**A little strength**	**No strength at all**
a. Arm that was most affected by your stroke?	5	4	3	2	1
b. Grip of your hand that was most affected by your stroke?	5	4	3	2	1
c. Leg that was most affected by your stroke?	5	4	3	2	1
d. Foot/ankle that was most affected by your stroke?	5	4	3	2	1

These questions are about your memory and thinking.

**2. In the past week, how difficult was it for you to...**	**Not difficult at all**	**A little difficult**	**Somewhat difficult**	**Very difficult**	**Extremely difficult**
a. Remember things that people just told you?	5	4	3	2	1
b. Remember things that happened the day before?	5	4	3	2	1
c. Remember to do things (e.g. keep scheduled appointments or take medication)?	5	4	3	2	1
d. Remember the day of the week?	5	4	3	2	1
e. Concentrate?	5	4	3	2	1
f. Think quickly?	5	4	3	2	1
g. Solve everyday problems?	5	4	3	2	1

These questions are about how you feel, about changes in your mood and about your ability to control your emotions since your stroke.

**3. In the past week, how often did you...**	**None of the time**	**A little of the time**	**Some of the time**	**Most of the time**	**All of the time**
a. Feel sad?	5	4	3	2	1
b. Feel that there is nobody you are close to?	5	4	3	2	1
c. Feel that you are a burden to others?	5	4	3	2	1
d. Feel that you have nothing to look forward to?	5	4	3	2	1
e. Blame yourself for mistakes that you made?	5	4	3	2	1
f. Enjoy things as much as ever?	5	4	3	2	1
g. Feel quite nervous?	5	4	3	2	1
h. Feel that life is worth living?	5	4	3	2	1
i. Smile and laugh at least once a day?	5	4	3	2	1

The following questions are about your ability to communicate with other people, as well as your ability to understand what you read and what you hear in a conversation.

**4. In the past week, how difficult was it to...**	**Not difficult at all**	**A little difficult**	**Somewhat difficult**	**Very difficult**	**Extremely difficult**
a. Say the name of someone who was in front of you?	5	4	3	2	1
b. Understand what was being said to you in a conversation?	5	4	3	2	1
c. Reply to questions?	5	4	3	2	1
d. Correctly name objects?	5	4	3	2	1
e. Participate in a conversation with a group of people?	5	4	3	2	1
f. Have a conversation on the telephone?	5	4	3	2	1
g. Call another person on the telephone, including selecting the correct phone number and dialing?	5	4	3	2	1

The following questions ask about activities you might do during a typical day.

**5. In the past 2 weeks, how difficult was it to...**	**Not difficult at all**	**A little difficult**	**Somewhat difficult**	**Very difficult**	**Could not do at all**
a. Cut your food with a knife and fork?	5	4	3	2	1
b. Dress the top part of your body?	5	4	3	2	1
c. Bathe yourself?	5	4	3	2	1
d. Clip your toenails?	5	4	3	2	1
e. Get to the toilet on time?	5	4	3	2	1
f. Control your bladder (not have an accident)?	5	4	3	2	1
g. Control your bowels (not have an accident)?	5	4	3	2	1
h. Do light household tasks/chores (e.g. dust, make a bed, take out garbage, do the dishes)?	5	4	3	2	1
i. Go shopping?	5	4	3	2	1
j. Do heavy household chores (e.g. vacuum, laundry or yard work)?	5	4	3	2	1

The following questions are about your ability to be mobile, at home and in the community.

**6. In the past 2 weeks, how difficult was it to...**	**Not difficult at all**	**A little difficult**	**Somewhat difficult**	**Very difficult**	**Could not do at all**
a. Stay sitting without losing your balance?	5	4	3	2	1
b. Stay standing without losing your balance?	5	4	3	2	1
c. Walk without losing your balance?	5	4	3	2	1
d. Move from a bed to a chair?	5	4	3	2	1
e. Walk one block?	5	4	3	2	1
f. Walk fast?	5	4	3	2	1
g. Climb one flight of stairs?	5	4	3	2	1
h. Climb several flights of stairs?	5	4	3	2	1
i. Get in and out of a car?	5	4	3	2	1

The following questions are about your ability to use your hand that was MOST AFFECTED by your stroke.

**7. In the past 2 weeks, how difficult was it to use your hand that was most affected by your stroke to...**	**Not difficult at all**	**A little difficult**	**Somewhat difficult**	**Very difficult**	**Could not do at all**
a. Carry heavy objects (e.g. bag of groceries)?	5	4	3	2	1
b. Turn a doorknob?	5	4	3	2	1
c. Open a can or jar?	5	4	3	2	1
d. Tie a shoe lace?	5	4	3	2	1
e. Pick up a dime?	5	4	3	2	1

The following questions are about how stroke has affected your ability to participate in the activities that you usually do, things that are meaningful to you and help you to find purpose in life.

**8. During the past 4 weeks, how much of the time have you been limited in...**	**None of the time**	**A little of the time**	**Some of the time**	**Most of the time**	**All of the time**
a. Your work (paid, voluntary or other)	5	4	3	2	1
b. Your social activities?	5	4	3	2	1
c. Quiet recreation (crafts, reading)?	5	4	3	2	1
d. Active recreation (sports, outings, travel)?	5	4	3	2	1
e. Your role as a family member and/or friend?	5	4	3	2	1
f. Your participation in spiritual or religious activities?	5	4	3	2	1
g. Your ability to control your life as you wish?	5	4	3	2	1
h. Your ability to help others?	5	4	3	2	1

9. Stroke Recovery

On a scale of 0 to 100, with 100 representing full recovery and 0 representing no recovery, how much have you recovered from your stroke?

_____ 100 Full Recovery

—

_____ 90

—

_____ 80

—

_____ 70

—

_____ 60

—

_____ 50

—

_____ 40

—

_____ 30

—

_____ 20

—

_____ 10

_______ 0 No Recovery

Item Clarifications

1. If patient says "I don't have an affected side", then instruct them to score using their perceived weaker side. If they still insist there is no affected, or weaker, side instruct them to score using their dominant side.

4. If patient says s/he does not do any or all of the items listed, code item(s) as *Extremely Difficult.*
   (Item f) If patient does not call but is handed the phone this is OK.
   (Item g) If patient cannot hold a phone book, if they can read it this is OK. This item addresses whether the patient is able to initiate a phone call, look up the number, and dial this number correctly.

5. If patient says s/he does not do any or all of the items listed, code item(s) as *Cannot do at all.*
   (Item a) If person is on pureed food, even if they feel they could cut the food, code as *Cannot do at All* (1/5/98)
   (Item c) Bathing oneself does not include getting into the tub.
   (Item e) This question is associated with movement. Does the person have the physical ability to get to the bathroom quickly enough?
   (Item f) Losing a little urine/dribbling is considered an accident.
      If person has intermittent catheter and is having no leaking problems code them as per report. (1/5/98)
      If person has an in-dwelling Foley catheter, code as *Cannot do at all.* (1/5/98)
   (Item g) Constipation is not counted here, person has to have an accident.
   (Item i) "Shopping" means any type of shopping and does not include driving.

6. If patient hasn't done any of the items in the past two weeks code as *Cannot do at all.*
   (Item h) If patient hasn't "climbed several flights of stairs" in two weeks, they may be prompted by saying "have you gone up and down one flight of stairs a couple of times in a row." If they still say they have not done it then they must be coded as *Cannot do at all.*
   (Item i) If the patient wants to know what kind of car say "your car" or "the car you ride in most."

7. If patient says "I don't have an affected side", then instruct them to score using their perceived weaker side. If they still insist there is no affected, or weaker, side instruct them to score using their dominant side.
   (Item a) If the patient says s/he has not been to the grocery store say "have you carried anything heavy with that hand."
   (Item d) This item is to tie a shoelace/bow using both hands.

8. If patient does not do any of the specific items (and has never done), code interference as *None of the time.*
